# Use of non‐LDL‐C lipid‐lowering medications in patients with type 2 diabetes

**DOI:** 10.1002/edm2.126

**Published:** 2020-04-14

**Authors:** Suzanne V. Arnold, Kensey Gosch, Nathan D. Wong, Vittal Hejjaji, Abhinav Goyal, Lawrence A. Leiter, Mikhail Kosiborod

**Affiliations:** ^1^ Saint Luke's Mid America Heart Institute and University of Missouri‐Kansas City Kansas City Missouri; ^2^ Irvine School of Medicine University of California Irvine California; ^3^ Emory University School of Medicine Atlanta Georgia; ^4^ Li Ka Shing Knowledge Institute St. Michael's Hospital University of Toronto Toronto Ontario Canada; ^5^ The George Institute for Global Health University of New South Wales Sydney New South Wales Australia

**Keywords:** diabetes mellitus, lipids, quality of care, triglycerides

## Abstract

**Background:**

A number of non‐low‐density lipoprotein cholesterol lipid abnormalities are associated with type 2 diabetes and insulin resistance, which may lead practitioners to use medications targeting these abnormal lipid fractions despite a lack of evidence or guideline recommendations.

**Methods and Results:**

Among 382 921 US patients with type 2 diabetes (69% with cardiovascular disease, 76% on a statin), 95 995 (26%) were on some nonstatin lipid‐lowering medication—19 265 (5%) on niacin, 32 919 (9%) on a fibrate and 69 513 (18%) on fish oil. Use of all three medications was stable over time and higher in patients with cardiovascular disease and with higher triglyceride levels, although even among patients with triglyceride levels <2.3 mmol/L, 6% were on a fibrate and 17% were on fish oil.

**Conclusion:**

As clinical trials demonstrate little to no cardiovascular benefit from taking these medications, greater attention is needed to focus the use of lipid‐lowering medications to those with proven benefit.

## Graphical abstract

A number of non‐low‐density lipoprotein cholesterol lipid abnormalities are associated with type 2 diabetes and insulin resistance, which may lead practitioners to use medications targeting these abnormal lipid fractions despite a lack of evidence or guideline recommendations. Among 382 921 US patients with type 2 diabetes (69% with cardiovascular disease, 76% on a statin), 95 995 (26%) were on some nonstatin lipid‐lowering medication—19 265 (5%) on niacin, 32 919 (9%) on a fibrate and 69 513 (18%) on fish oil. Use of all three medications was stable over time and higher in patients with cardiovascular disease and with higher triglyceride levels, although even among patients with triglyceride levels <1.7 mmol/L, 5% were on a fibrate and 16% were on fish oil. As clinical trials demonstrate little to no cardiovascular benefit from taking these medications, greater attention is needed to focus the use of lipid‐lowering medications to those with proven benefit.

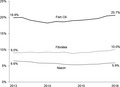

## 1 INTRODUCTION

Type 2 diabetes mellitus (T2D) is associated with a number of lipid abnormalities, including high triglycerides, low high‐density lipoprotein cholesterol (HDL‐C), increased low‐density lipoprotein cholesterol (LDL‐C) oxidation and glycation^1^—all of which increase the risk of atherosclerotic cardiovascular disease (ASCVD).^2^ While statins remain the key lipid‐lowering medications to reduce the risk of atherosclerosis‐related complications in patients with diabetes,^3^, ^4^, ^5^ lipid‐modifying therapies that primarily impact triglycerides and HDL‐C have also been commonly used in patients with diabetic dyslipidaemia. However, the evidence for the use of drugs that target these lipid fractions is substantially less robust than medications that lower LDL‐C,^6^, ^7^, ^8^ although notably one recent trial showed significant cardiovascular benefit with icosapent ethyl (a type of omega‐3 fatty acid that lowers triglycerides).^9^ For the time being, both US and European diabetes practice guidelines recommend medical therapy for hypertriglyceridaemia to be reserved for patients with substantially elevated triglycerides (>2.3 mmol/L) to reduce the risk of pancreatitis and not for ASCVD risk reduction,^10^, ^11^ given the negative trial results for fibrates^6^, ^7^ and fish oil.^8^, ^12^ Furthermore, niacin as a treatment for low HDL‐C is no longer recommended given the negative results of two large clinical trials.^13^, ^14^ It is unknown how these medications are currently used in patients with T2D—both for primary and secondary prevention of ASCVD. Given the unique aspects to diabetic dyslipidaemia and the underlying atherosclerotic risk of patients with diabetes, exploring the contemporary use of non‐LDL‐C lipid medications in these patients may provide important insights.

## 2 METHODS

### 2.1 Patient population

The data source for this analysis was the Diabetes Collaborative Registry (DCR), a US quality improvement registry designed to describe the outpatient care of diabetes through the spectrum of primary and specialty care.^15^ Patient data, including laboratory data, are extracted from electronic health records from 2014 to 2016 with the most recent visit used. We restricted the analysis to patients with T2D and available lipid levels. Because registry participation requires no data collection beyond that of the routine clinical care and due to the de‐identified nature of the collected information, waiver of written informed consent and authorization for this study was granted by Chesapeake Research Review Incorporated.

### 2.2 Statistical analysis

We examined the use of fibrates, niacin and fish oil (documented in the health record) in the following groups: overall, ASCVD (coronary artery disease, peripheral artery disease, cerebrovascular disease) versus no ASCVD, statin versus no statin, according to different triglyceride levels, and over time. The last patient visit was used for analysis. To examine use over time, the last patient visit in each quarter was used. We compared demographics, comorbidities and lipid levels for those patients on versus not on each of the medications using standardized differences (>10% difference is considered clinically relevant). All analyses were performed with SAS version 9.4 (SAS Institute).

## 3 RESULTS

**Figure 1:**
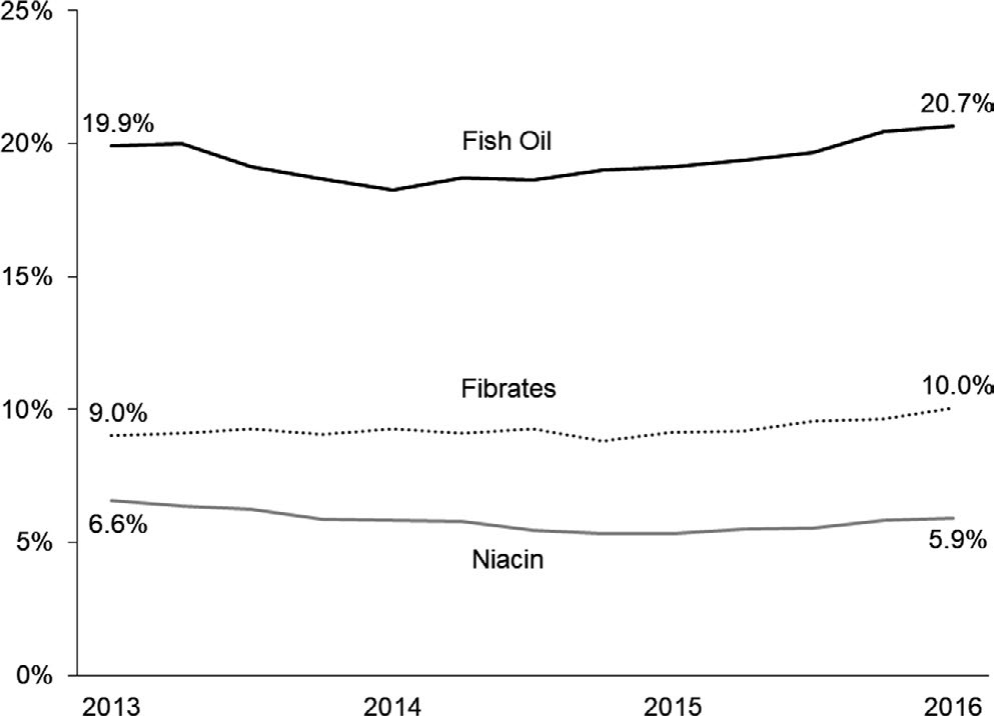
Use of non‐LDL‐C lipid medications over time

Among 942 269 adults with T2D enrolled in DCR, lipid levels were available for 382 921 patients (40.6%), who formed the analytic cohort. Patients with lipid data were more likely to be older and with ASCVD (Table S1). Mean age of the analytic cohort was 68.5 ± 11.7 years, 56.6% were men, mean body mass index was 32.0 ± 6.9 kg/m^2^, and 68.7% had established ASCVD (Table 1). Mean cholesterol was 4.2 ± 1.1 mmol/L, triglycerides were 1.7 ± 1.0 (median 1.5, IQR 1.1‐2.1), HDL‐C was 1.2 ± 0.4 mmol/L, and LDL‐C was 2.2 ± 0.9 mmol/L. Overall, 289,390 patients (75.6%) were on a statin and 95,995 patients (25.9%) were on some nonstatin lipid‐lowering medication—19 265 patients (5.0%) on niacin, 32 919 (8.6%) on a fibrate and 69 513 (18.2%) on fish oil (Table S2). Use of all three medications was higher in patients with versus without ASCVD, on versus not on a statin and with higher triglyceride levels (Table 1). There have been only small changes in the use of all three medications over time (Figure 1), with use of niacin decreasing 11%, fibrates increasing 11% and fish oil increasing 4% from 2013 to 2016.

**Table 1:** Patient characteristics

	All patients n = 382,921	Niacin n = 19,265	Fibrate n = 32,919	Fish oil n = 69,513
Age, years	68.5 ± 11.7	69.3 ± 10.0	67.3 ± 10.6^a^	69.6 ± 10.3^a^
Men	56.6%	74.6%^a^	64.5%^a^	61.0%^a^
Body mass index, kg/m^2^	32.0 ± 6.9	31.8 ± 6.2	32.7 ± 6.4^a^	31.9 ± 6.5
HbA1c, %	7.3 ± 2.0	7.1 ± 1.9	7.5 ± 2.0^a^	7.1 ± 1.8^a^
Glucose‐lowering medications	65.7%	75.2%^a^	82.8%^a^	72.6%^a^
Insulin	18.6%	22.7%^a^	29.9%^a^	21.5%^a^
ASCVD	68.7%	82.6%^a^	75.7%^a^	75.6%^a^
Coronary artery disease	60.9%	76.6%^a^	68.1%^a^	67.9%^a^
Peripheral artery disease	17.9%	21.3%	21.0%	19.5%
Prior stroke	12.0%	17.3%^a^	15.0%	15.4%^a^
Heart failure	25.9%	26.4%	25.7%	24.8%
Atrial fibrillation	23.4%	22.6%	22.0%	23.8%
Chronic kidney disease	9.6%	10.3%	13.4%^a^	10.5%
Current smoker	14.3%	14.7%	15.8%	12.2%
Statin	75.6%	86.5%^a^	82.2%^a^	83.5%^a^
Total cholesterol, mmol/L	4.2 ± 1.1	4.0 ± 1.1^a^	4.2 ± 1.1	4.1 ± 1.1
HDL cholesterol, mmol/L	1.2 ± 0.4	1.1 ± 0.4^a^	1.0 ± 0.3^a^	1.2 ± 0.4
LDL cholesterol, mmol/L	2.2 ± 0.9	2.0 ± 0.9^a^	2.1 ± 0.9^a^	2.1 ± 0.9^a^
Triglycerides, mmol/L	1.7 ± 1.0	1.8 ± 1.1^a^	2.3 ± 1.3^a^	1.9 ± 1.1^a^
<1.7 mmol/L	60.0%	55.4%^a^	37.5%^a^	54.3%^a^
1.7‐2.2 mmol/L	19.0%	19.5%^a^	21.5%^a^	20.0%^a^
2.3‐5.6 mmol/L	20.3%	23.9%^a^	38.3%^a^	24.6%^a^
>5.6 mmol/L	0.7%	1.2%^a^	2.8%^a^	1.1%^a^

Note

ASCVD, atherosclerotic cardiovascular disease (ie coronary artery, peripheral artery or cerebrovascular artery disease); HDL, high‐density lipoprotein; LDL, low‐density lipoprotein.

^a^ Indicates standardized difference >10% in comparison with those patients not on the particular medication.

## 4 DISCUSSION

In a large cohort of patients with T2D, we found that lipid levels were not markedly abnormal. Non‐LDL‐C lipid‐lowering medications were not infrequently used in patients with T2D, with an even greater use in those with established atherosclerosis. Furthermore, in patients with triglyceride levels <1.7 mmol/L, we found that 5% were on a fibrate and 16% were on fish oil and 5% of all patients were on niacin. Finally, we found general stability in these rates over time, indicating that practice patterns do not appear to be rapidly changing. Greater attention is needed to focus lipid‐lowering strategies to those with proven cardiovascular benefit.

A prior analysis examined use of nonstatin lipid‐lowering medications in elderly patients with coronary artery disease from 2007 to 2011 and found that 5% of patients were on fibrates and 3% were on niacin.^16^ Patients with diabetes were more likely to be treated with a nonstatin lipid‐lowering medication, although this analysis grouped all nonstatin medications together (including ezetimibe and bile acid sequestrants). Furthermore, this study showed fairly stable rates of fibrate and niacin use over 2007‐2011, despite the fact that the pivotal trials showing overall lack of cardiovascular benefit with these medications were published during this time frame. The rates in this study were lower than what we observed in our DCR cohort, which likely reflects the lipid abnormalities associated with diabetes. Interestingly, we also observed stable rates in use from 2013 to 2016, indicating that knowledge regarding the appropriate use of these medications with available evidence base has yet to penetrate routine clinical practice.

There are limitations to our analysis that merit further discussion. First, we do not have pretreatment lipid levels to know whether a patient's triglycerides were markedly elevated, making treatment with fish oil and/or fibrates appropriate. Second, while over‐the‐counter fish oil and niacin are often documented in electronic health records, we cannot know whether a patient was taking these supplements without the knowledge of the physician. Third, fibrates appear to have some benefit in reducing nonfatal cardiovascular events in statin‐intolerant patients, and fish oil has previously been recommended in cardiology guidelines for secondary prevention of coronary artery disease. As such, some patients with established ASCVD were likely given these medications for these reasons. Furthermore, with publication of the trial with high‐dose icosapent ethyl^9^ and ongoing studies of fibrates with statins in high‐risk patients, guidelines may again change for patients with ASCVD and elevated triglycerides. Fourth, our cohort had a high prevalence of ASCVD, which was associated with higher use of all 3 classes of non‐LDL‐C lipid‐lowering medications. As such, overall use numbers would be expected to be lower in a general T2D population. Finally, we cannot document harm from any apparent overtreatment with nonstatin lipid‐lowering medications.

In conclusion, we found that medications that target non‐LDL‐C lipid fractions are commonly used in patients with T2D, which likely reflects the underlying lipid abnormalities associated with T2D/insulin resistance. As clinical trials have thus far demonstrated little cardiovascular benefit from taking these medications, greater attention is needed to focus the use of lipid‐lowering medications to those with proven benefit.

## CONFLICT OF INTEREST

NDW received research support through institution from Amgen, Boehringer Ingelheim, Amarin, Novo Nordisk; advisory board from Novaris, Amarin; speaker fees from Sanofi, Amarin; and consulting honoraria from Boehringer Ingelheim. LAL received research grants from Amgen, Esperion, Kowa, Regeneron/Sanofi, The Medicines Company and personal fees from Amgen, HLS, Kowa, Merck, Regeneron/Sanofi, The Medicines Company. MK received research grants from AstraZeneca, Boehringer Ingelheim; other research support from AstraZeneca; and consulting honoraria from AstraZeneca, Amgen, Bayer, Boehringer Ingelheim, Novo Nordisk, Sanofi, GSK, Merck (Diabetes), Eisai, Intarcia, Novartis and Glytec. The remaining authors report no relevant disclosures to the current manuscript.

## AUTHORS' CONTRIBUTIONS

SVA, KG, NDW, VH, AG, LAL and MK contributed to design of the work; SVA, NDW and MK contributed to data acquisition; KG and SVA contributed to data analysis; SVA, KG, NDW, VH, AG, LAL and MK contributed to interpretation of analyses; SVA contributed to drafting the work; KG, NDW, VH, AG, LAL and MK contributed to critical revisions.

## Supporting information

Table S1‐S2

## Data Availability

The data that support the findings of this study are available from the Diabetes Collaborative Registry, which is managed by the American College of Cardiology. Restrictions apply to the availability of these data.
