# Supplementary material for: Use of non‐LDL‐C lipid‐lowering medications in patients with type 2 diabetes
Source: Endocrinol Diabetes Metab. 2020 Apr 14;3(3):e00126. doi: 10.1002/edm2.126 (PMC7375067; doi:10.1002/edm2.126)
Supplement: Supplementary file 1 — Table S1‐S2 [file EDM2-3-e00126-s001.docx]

| **Supplemental Table 1. Comparison of patients with versus without lipid data** | | | |
| --- | --- | --- | --- |
|  | **Lipid Data n=382,921** | **No Lipid Data n=559,348** | **Standardized Difference** |
| Age, years | 68.5±11.7 | 67.2±12.8 | 12.1% |
| Men | 56.6% | 52.7% | 8.0% |
| Body mass index, kg/m^2^ | 32.0±6.9 | 32.3±7.4 | 5.0% |
| HbA1c, % | 7.3±2.0 | 7.8±2.4 | 22.5% |
| Glucose-lowering medications | 65.7% | 68.2% | 5.3% |
| Insulin | 18.6% | 20.0% | 1.9% |
| ASCVD | 68.7% | 60.1% | 19.2% |
| Coronary artery disease | 60.9% | 51.6% | 19.7% |
| Peripheral artery disease | 17.9% | 14.1% | 11.6% |
| Prior stroke | 12.0% | 11.5% | 3.2% |
| Heart failure | 25.9% | 23.9% | 6.6% |
| Atrial fibrillation | 23.4% | 21.8% | 5.1% |
| Chronic kidney disease | 9.6% | 7.5% | 8.9% |
| Current smoker | 14.3% | 15.4% | 3.5% |

ASCVD, atherosclerotic cardiovascular disease

| **Supplemental Table 2. Use of non-statin lipid-lowering medications** | | | | | | | |
| --- | --- | --- | --- | --- | --- | --- | --- |
|  |  | **Niacin** | | **Fibrates** | | **Fish Oil** | |
|  | **N** | **Use** | **Std Diff** | **Use** | **Std Diff** | **Use** | **Std Diff** |
| Overall | 382,921 | 5.0% |  | 8.6% |  | 18.2% |  |
| Atherosclerotic cardiovascular disease |  |  | 15.9% |  | 10.2% |  | 15.6% |
| Yes | 262,977 | 6.0% |  | 9.5% |  | 20.0% |  |
| No | 119,944 | 2.8% |  | 6.7% |  | 14.1% |  |
| On statin |  |  | 14.8% |  | 11.6% |  | 21.3% |
| Yes | 289,390 | 5.8% |  | 9.4% |  | 20.1% |  |
| No | 93,531 | 2.8% |  | 6.3% |  | 12.3% |  |
| Triglyceride level |  |  |  |  |  |  |  |
| <1.7 mmol/L | 229,915 | 4.6% |  | 5.4% |  | 16.4% |  |
| 1.7-2.2 mmol/L | 72,717 | 5.2% | 2.5% | 9.7% | 16.6% | 19.1% | 7.1% |
| 2.3-5.6 mmol/L | 77,554 | 5.9% | 5.8% | 16.2% | 35.6% | 22.1% | 14.4% |
| >5.6 mmol/L | 2,735 | 8.2% | 14.4% | 33.3% | 75.6% | 28.7% | 29.7% |
| HDL-C level |  |  |  |  |  |  |  |
| <1.03 mmol/L | 138,612 | 6.4% |  | 13.1% |  | 19.6% |  |
| 1.03-1.55 mmol/L | 184,246 | 4.1% | 10.1% | 6.5% | 22.3% | 17.1% | 6.6% |
| >1.55 mmol/L | 52,507 | 3.3% | 14.1% | 3.5% | 35.5% | 16.6% | 7.9% |

For triglycerides and HDL-C, standardized differences compare use in each group compared with the lowest
